# Mathematical Modeling of Yarn Dynamics in a Generalized Twisting System

**DOI:** 10.1038/srep24432

**Published:** 2016-04-15

**Authors:** R. Yin, X. M. Tao, B. G. Xu

**Affiliations:** 1 Institute of Textiles and Clothing, The Hong Kong Polytechnic University, Hong Kong, China

## Abstract

Twisting is an important process to form a continuous yarn from short fibres and to determine the structure and properties of the resultant yarn. This paper proposes a new theoretical model of yarn dynamics in a generalized twisting system, which deals with two important phenomena simultaneously, that is, twist generation and twist propagation. Equations of yarn motion are established and the boundary value problems are numerically solved by Newton-Raphson method. The simulation results are validated by experiments and a good agreement has been demonstrated for the system with a moving rigid cylinder as the twisting element. For the first time, influences of several parameters on the twisting process have been revealed in terms of twist efficiency of the moving rigid cylinder, propagation coefficients of twist trapping and congestion. It was found that the wrap angle and yarn tension have large influence on the twisting process, and the yarn torsional rigidity varies with the twisting parameters.

## Introduction

Twisting is a key process of making a continuous yarn^1^ from short discontinuous fibers, such as carbon nanotubes, cotton or wool etc. It strongly influences the structure, mechanical strength, rigidity, thermal and electric conductivity as well as surface characteristics of the resultant yarn. The yarns are then woven or knitted into fabrics for apparel and home-textile applications^2^. Recently, carbon nanotubes or functional fibres have been employed to manufacture smart yarns for sensing^3^,^4^,^5^,^6^,^7^, actuating^8^,^9^,^10^,^11^, and energy harvesting^12^,^13^,^14^,^15^,^16^ by the resemble spinning principle. Similarly, during the formation of continuous composite fibers with one-dimensional fillers like nanotubes and liquid crystals, twisting may introduce a desired orientation and distribution of the fillers in the fibers^2^.

**Figure 1:**
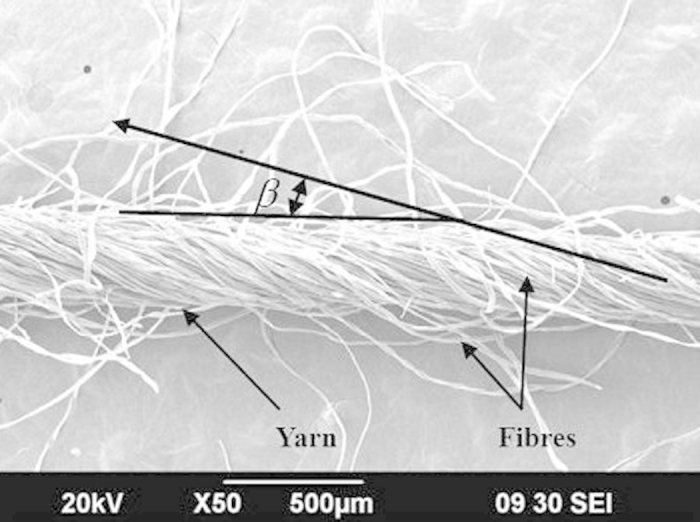
SEM micrograph of a 20Ne cotton yarn.

In all these yarns, the majority of the surface fibres follow a helical path with a helix angle β with respect to the yarn direction, as shown in the SEM micrograph of Fig. 1,


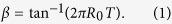


where *R*_*0*_ is the yarn radius and *T* is the inserted number of twists per unit length of yarn.

Twisting determines the yarn helical structure, and performances such as strength, elongation, evenness and hairiness, by manipulating a bundle of separated short fibres and assembling them into a consolidated yarn^17^,^18^,^19^. In most textile manufacturing processes, the number of twist in yarn varies along its length because of the positive twisting torque generated by the twisting element or twist blockage by contacting with the surface of a machine part^20^. Much valuable work has been carried out to investigate the twisting processes in ring spinning^21^,^22^,^23^,^24^,^25^, rotor spinning^26^,^27^,^28^, friction spinning^29^, self-twist spinning^30^, air-jet spinning^31^, etc. Meanwhile, the twist blockage caused by a yarn guide^32^ or tension meter^33^,^34^ in spinning and weaving/knitting machines has been widely explored. However, few studied the situation where the twisting generation and twist blockage during propagation coexist on single machine part.

Recently, a novel twisting system has been developed by introducing a moving rigid cylinder^35^, which is incorporated in a ring spinning machine as a false-twisting element, and at the same time blocks the twist propagation generated by the spindle and by the false-twisting element. The treatment of a moving rigid cylinder can be applicable to various problems, for instance, reducing the twist blockage in the twisting or roving machine to enhance the spinnability^36^, as well as modifying the yarn internal structure, such as reducing the yarn residual torque^37^, altering the porosity or diameter of the yarns for functional activities^38^,^39^,^40^.

Apparently, it is necessary to develop a theoretical model for the dynamic performance of the yarn motion on a moving rigid cylinder. In the present study, both twisting generation and twist blockage are considered in the novel twisting system. Dynamic equations of a moving yarn are established and solved numerically by Newton-Raphson method. The simulation results are validated experimentally by using high-speed photography and tension measurement. Finally, influences of various system parameters on the twist efficiency of the moving rigid cylinder, the propagation coefficients of twist trapping and congestion are identified.

### Twisting efficiency, twist trapping and twist congestion in a generalized twisting system

**Figure 2:**
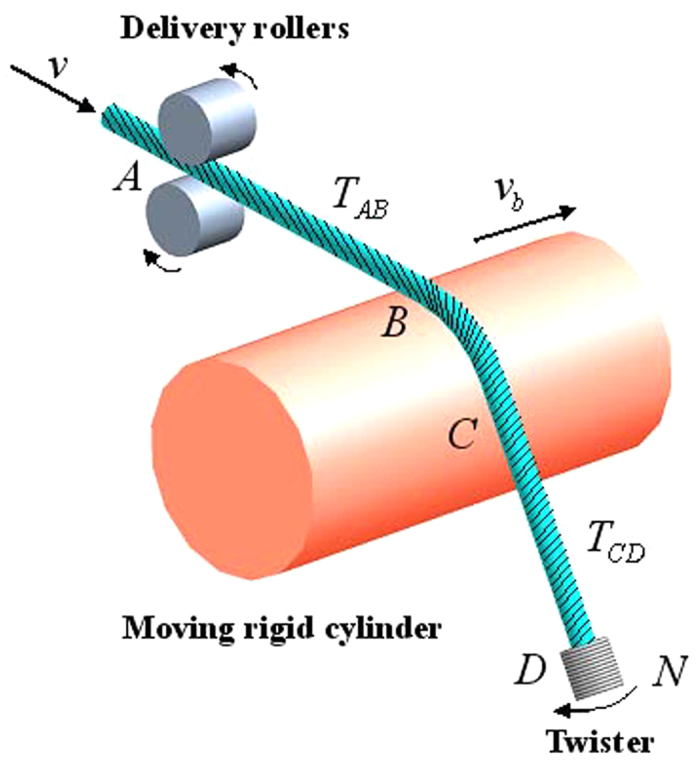
A generalized twisting system.

As shown in Fig. 2, the twisting system is composed of delivery rollers at point A, a translationally moving rigid cylinder, which contacts with the yarn in zone BC and a twister at point D. Hence, there are two twisters in the system: one is the real-twister at point D; another is the false-twister which generates the torque by the frictional moment at zone BC. During the spinning process, the yarn moves at a constant velocity *v*, but its twist level is altered in different zones. The bifurcations or instabilities of twisted yarn, as twisted elastic rod, under specific conditions^42^,^43^ are unlikely to occur in our study. Torsional bifurcations are prohibited to happen because the levels of tension and twist are controlled below the critical value. Thus the twisting process is stable.

In order to describe the processes of this twisting system, three concepts are introduced to describe the roles played by friction, correspondingly, three coefficients are defined. The first concept is the twisting efficiency of the moving rigid cylinder. At the contacting area of the yarn surface and the rigid cylinder, the frictional moment forces the yarn to rotate along its axis. If there is no slippage or jumping of the yarn on the moving cylinder, then the yarn’s tangential velocity and the moving velocity of the cylinder at the contacting point should be the same. In this situation, the twist efficiency of the rigid cylinder is unity. Normally the twist efficiency is a value close to but below the unity. To determine it, let *R*_0_ be the radius of the yarn, *n*_*c*_ = *T*_*c*_*v* be the rotational speed of the yarn, *T*_*c*_ be the total twist generated by the moving rigid cylinder, *v* be the delivery speed of the yarn, and *v*_*b*_ be the moving speed of the rigid cylinder, the twist efficiency of the moving rigid cylinder is expressed as


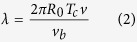


The second effect is the twist trapping in the up-ward propagation of the real twist inserted by D. Without the existence of the moving cylinder, the yarn twist generated by the real-twister at point D can be freely propagated into zone AB. Due to the introduction of the moving cylinder, a certain proportion of this twist is blocked because of the frictional moment generated in zone BC. To quantify this effect, let *T*_*t*_ be the total twist lost in zone BC, and *T*_*CD*_ be the twist in zone CD, then the propagation coefficient of twist trapping is defined as


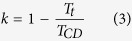


The last effect of friction is the twist congestion, which occurs in the downward propagation of twist in zone AB. The downward travelling yarn has a twist thus a tendency to untwist in zone BC. It is subject to another frictional moment, as a result, the rotating trend of the yarn is reduced, which blocks the yarn twist propagating into zone CD. The result is that the yarn twist is increased in zone AB. Let *T*_*h*_ be the total twist increment in zone AB, then the propagation coefficient of the twist congestion is defined as


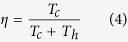


From the kinematic point of view, the twist in zone AB in Fig. 2 can be expressed as


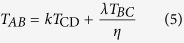


where 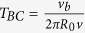 is the theoretical twist generated by the moving rigid cylinder, and all three coefficients range from 0 to 1.

The similarities of the twist trapping and twist congestion of the false-twister are: 1) Both are caused by friction. 2) They block the twist propagation. 3) They will not change the final yarn twist. The difference between the two is that in trapping, the tangential component of the frictional force reduces twist to the yarn; while in congestion, the tangential component of the friction force adds twist to the yarn. Thus, the functions of the friction force are to simultaneously insert twist in zone AB, congest the generated twist downwards, and trap the twist generated by the twister D upwards. In the steady state, there is no twist-gain from the moving rigid cylinder in zone CD because the frictional moment generates identical number of twists in each of the two zones, but in opposite directions. Therefore, when the yarn moves, the two parts offset each other, resulting in zero twist-gain in zone CD.

### Equations of motion

#### Assumptions

Several assumptions are made in the analysis: 1) The yarn is assumed to be inextensible since twisting of yarn is the dominating phenomenon, the deformation in the yarn axial direction is small thus can be ignored; 2) The yarn is assumed to have small bending moment during the process. This assumption is made based on the fact that yarn twist used in this study is at least one order of magnitude higher than that of the yarn bending curvature, although the bending and torsional stiffness of the yarn are of the same order^44^,^45^. The resultant torque is much higher than the moment generated by bending against the moving cylinder, therefore, it is reasonable to omit the bending effect; 3) A uniform yarn is assumed with a single linear density and cross-sectional area; 4) The weight of yarn is relatively small when compared with other forces thus can be neglected; 5) The moving cylinder has much greater rigidities thus can be regarded as non-deformable; 6) The moving cylinder is smooth with a constant curvature radius; 7) A linear relationship between the yarn twist and torque is assumed according to previous experimental results reported^46^,^47^; 8) The model is built in a steady state, thus time-dependent terms in the equations are ignored; 9) The study deals with stable twisting processes where no mechanical instability or bifurcation occurs.

#### Coordinate systems

**Figure 3:**
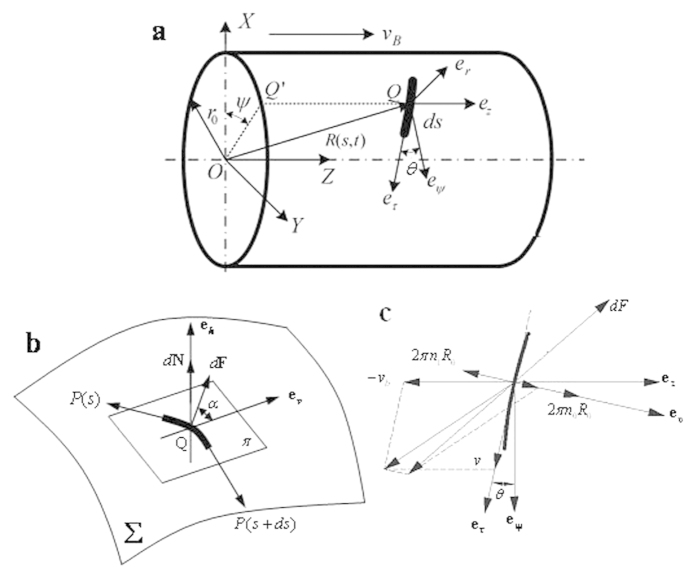
Modelling analysis of a yarn segment. (**a**) A yarn segment in the moving coordinate system. (**b**) Forces acting on a yarn element. (**c**) Analysis on yarn motion.

Considering an arbitrary point **Q** of the yarn, which at time *t* is at a distance *s* measured along the yarn from the initial contacting point **A (***s* = *0*), as shown in Fig. 3a. For the convenience of analysis, a fixed cylindrical coordinate system is selected with base vectors **e**_**r**_, **e**_**ψ**_, **e**_**z**_. The origin of coordinate **O** coincides with the centre of the initial contacting surface, and the z axis of the system is in line with the central axis of the rigid cylinder with its positive direction towards the moving direction. Let *r*_0_, *ψ, z* be the cylindrical coordinates corresponding to the coordinate frame and **R**(*s*,*t*) = *r*_0_**e**_*r*_ + *z***e**_*z*_be the position vector of Q relative to the origin O.

In order to simplify the derivation, a moving coordinate frame with its base unit vectors **e**_*τ*_, **e**_*h*_, **e**_*v*_ is introduced. In Fig. 3b, *π* is the plane tangent to the moving surface at point Q. The unit vectors **e**_*τ*_ and **e**_*h*_ are that in the direction of yarn motion and that normal to the tangent plane *π*, respectively, and **e**_*v*_ is expressed as **e**_*v*_ = **e**_*h*_ × **e**_*τ*_. *θ* is the angle formed between **e**_*τ*_ and **e**_*ψ*_. In this analysis, we define that *θ* is positive when **e**_*ψ*_ is at the right side of **e**_*τ*_, negative when **e**_*ψ*_ is at the left side of **e**_*τ*_.

In a cylindrical coordinate system (*r*_0_, *ψ, z*), **e**_*τ*_, **e**_*h*_, **e**_*v*_ can be written as


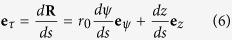



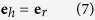



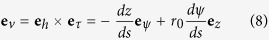


Also, the second derivative of **R** with respective to *s* can be expressed in the same manner as


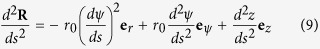


#### Force balance

If **P**(*s, t*) is the tension in the yarn at **Q**, **N**(*s, t*) is the normal reaction force, and **F**(*s, t*) is the friction force, then the full vector form of the time-dependent equation of motion for yarn at **Q** is^21^


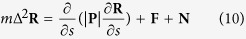


where *m* is the linear density of the yarn. The differential operator △ is given by 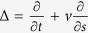, where *v* is the constant delivery speed of the yarn. In the steady state, the solution of equation (10) is independent of time, and the operator reduces to 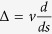. Therefore, equation (10) can be rewritten as


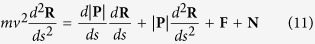


The unit normal reaction force **N,** in the moving coordinate system, can be expressed as


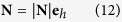


The friction force acting on the yarn as it slides over the rigid cylinder follows the Coulomb friction law


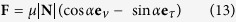


where *μ* is the friction coefficient between the yarn and cylindrical surface, *α* is the friction angle between the direction of friction force and the unit vector **e**_*v*_.

The yarn speed on the rigid cylinder is composed of three components: yarn rotational speed around its own axis 2*πR*_0_(*n*_1_ − *n*_0_)**e**_*v*_, yarn delivery speed *v***e**_*τ*_, and moving speed of surface *v*_*b*_**e**_*z*_, as shown in Fig. 3c, where *n*_0_ is the rotational speed of the yarn generated by the twister, and *n*_1_ is the rotational speed of the yarn generated by the moving surface.

Thus, the angle *α* can be derived as


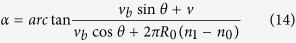


The inextensible condition gives


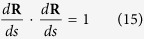


Therefore, the following equation can be obtained


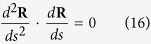


The scalar formulas of equation (11) in the moving coordinate system can be rewritten after some rearrangement as follows,


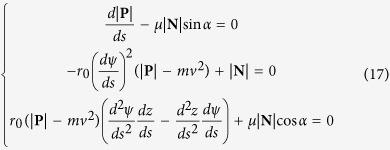


According to the analysis of differential geometry in Fig. 3a, one can obtain


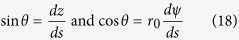


And the following equations can be derived


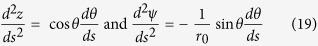


Substituting equations (18) and (19) into equation (17), eliminating the unit normal reaction force **N**, the two independent first-order differential equations are obtained


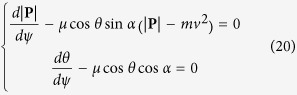


#### Moment balance

As shown in Fig. 3b, the moment equilibrium^17^ for a yarn element can be derived as


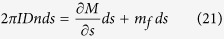


where *I* is the moment of inertia per unit length of yarn, *n* is the rotational speed of yarn around its own axis, *M* is the yarn torque, *m*_*f*_ is the external moment and in the case of the friction moment, *m*_*f*_ = *μ*|**N**|cos *αR*_0_, in which *R*_*0*_ is the radius of yarn.

In the steady state, equation (21) becomes


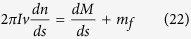


Since a linear relationship is assumed between the twist and torque (*M* = *KT*), together with the twist flow equilibrium 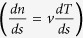, equation (22) can be further expressed as


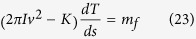


Substituting equation (18) into equation (23), the following equation can be obtained.


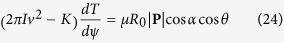


### Calculating the coefficients of twist efficiency and twist blockage

Substituting and rearrangement of equations (14) and (24) yield:


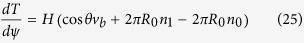


where 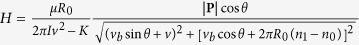


Equation (25) shows that yarn twist variation is depended on three factors: the first factor contains the velocity component of the moving rigid cylinder, the second and third factors contain the rotational speeds of the yarn generated by the false-twister and real-twister, respectively

Integrating equation (25) into the following


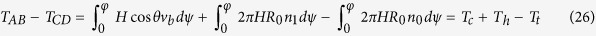


Replacing *T*_*c*_, *T*_*h*_ and *T*_*t*_ into equations (2, 3, 4), and the three key coefficients are given by


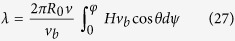



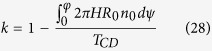



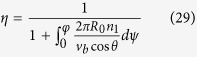


### Boundary equations

This system is composed of three first-order differential equations (20 and 24). In addition, the rotational speed *n*_1_ and the torsional rigidity *K* are two unknown constant values. Therefore, totally five boundary conditions are needed to make this problem solvable.

One boundary equation can be derived based on the geometrical condition. The delivery rollers at the point A and the twister at the point D are in the same plane that parallels to the XOY plane. The length of line AB and CD are at least one order of magnitude higher than that of curve BC. Therefore, the deviation angles for line AB and CD follow,


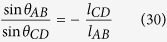


Another boundary equation is directly obtained from the kinematic formula. Multiplying delivery speed *v* in equation (2) and replacing *n*_1_ by *λT*_*BC*_*v*, and rearranging the equation lead to


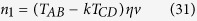


The other three boundary values are |**P**|_AB_, *T*_AB_ and *T*_CD_, which were obtained from the measurements using the high-speed camera and tension meter systems.

### Dimensionless equations

The convenient scales for the dimensionless analysis are surface radius *r*_0_ for length, yarn delivery speed *v* for speed, yarn tension |**P**|_AB_ for force per unit length, yarn twist *T*_*CD*_ for twist. All variables are normalized in a dimensionless form as follows


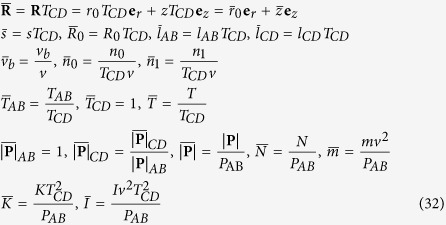


Since 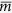 and 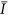 are small terms in the equations, they can be omitted without losing the accuracy of the solutions. The dimensionless equations of force equilibrium and twist distribution become


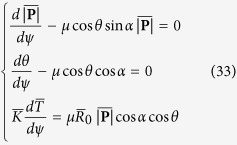


where 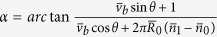


The dimensionless forms of the boundary conditions are


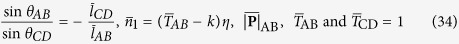


### Method of numerical solution

The finite difference method for the numerical solution was applied to solve the equations presented in this paper with Matlab. The transformed equation (33) were integrated numerically over the domain 0 ≤ *ψ* ≤ *φ*. First, the wrap angle *φ* was divided into *n* small segments, and each segment had an equal degree *φ*/*n*. Then, the variables 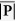, *θ*, 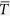 were discretized as follows,


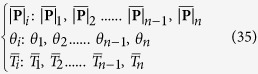


and replacing the derivatives with respect to *ψ* by central difference operators,


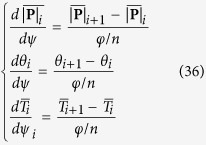


Therefore, the differential equations were simplified to algebraic equations as follows,


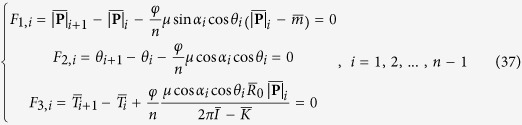


The boundary conditions were also transformed below,


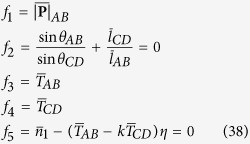


The solutions were found by the following scheme: First, initialize the known parameters and input the three boundary values from the experimental measurement. Next, create trial matrix **X**_0_ which was composed of unknown variables 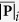, *θ*_*i*_, 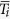, and unknown constant value *K*. Then, create trial values for *λ, k, η*, thus, 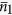 was calculated using equation (31). After that, Jacobian matrix was generated and iterated by the Newton-Raphson scheme until the norm of the functions was smaller than 1e^−5^. If the results of two adjacent iteration for 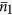 was larger than 1e^−5^, use the new 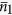 as trial values for iteration. Finally, the three unknown variables and two unknown constant values were obtained, and *λ, k, η* were computed.

### Experimental setup

The experiments were conducted on a ring spinning frame (Zinser 351) by installing a moving rigid cylinder with diameter of 6mm between the front rollers and the yarn guide. A cotton yarn with linear density of 18.45 g/km and diameter of 0.16 mm was adopted for the measurement. The experimental yarn and its measurement instrumentation are described in details by Feng *et al*.^41^. The measurement system was composed of two parts. One was for online acquisition of yarn tension, which included a strain gauge sensor (Honigmann tension meter 125.12, 100cN maximum range, 0.1cN precision, 15° measuring angle), A/D converter (ZTIC USB-7660, digital I/O, USB form factor, 48 channels analog input, 12 bits size, 50KHz maximum clock rate, 0–10 V voltage range, 5 MΏ input impedance) and computer software (NI Labview 7.5). The second part was for continual and automatic image acquisition, storage and analysis of yarn instant twist including a high-speed camera (Phantom MIRO 4, CMOS sensor, 800 × 600 pixels, over 1200 fps at full resolution, 22 μm pixel size, 12-bit depth), which was connected to a personal computer installed with camera control software and Nikon micro lens (AF Micro-Nikkor 60 mm f/2.8D). Using the system, all data were acquired under the above mentioned conditions, ensuring repeatability. The details of the calibration process are given in Supplementary Information S1. In addition, the frictional coefficient of the yarn and the rigid cylinder was measured on the Shirley friction meter. In the following analysis, tension |**P**|_*AB*_, twist *T*_*AB*_, and *T*_*CD*_ were measured as the boundary conditions to solve the theoretical model, while tension |**P**|_*CD*_, deviation angle *θ*_*AB*_ and *θ*_*CD*_ were measured to verify the accuracy of the theoretical model.

## Results and Discussion

### Verification of model

The dynamic model was verified before further investigation. Three cases with different operational parameters were studied, as shown in Table 1. After numerical simulation, the results of |**P**|_*CD*_, *θ*_*AB*_ and *θ*_CD_ were theoretically obtained and then compared with experimental measurements for verification.

The moving cylinder was installed at the middle of the delivery rollers and the twister. 32 Ne black-white yarn was used for the experiments. The radius of the yarn was 0.08 mm, and the frictional coefficient of the yarn and moving cylinder was 0.81. Based on the parameters given in Table 1, simulation results of distributions of yarn twist, tension and deviation angle on the moving surface for the three cases were obtained. Table 1 lists the simulated and measurement values of yarn tension and deviation angle. In all three cases, the difference between simulated values and experimental observations is smaller than 10%, which implies that the simulated figures match well with the measurement values and the theoretical model can predict a relatively accurate value of the problem. Additionally, the variation of tension measurement is smaller than 10%, while that for the measured twist and deviation angle are as large as 15.97% and 15.87% in case 2 and case 3, respectively. The large variations of yarn twist and deviation angle are mainly caused by the relative motion of the yarn on the moving surface.

### Distributions of tension, deviation angle and twist

**Figure 4:**
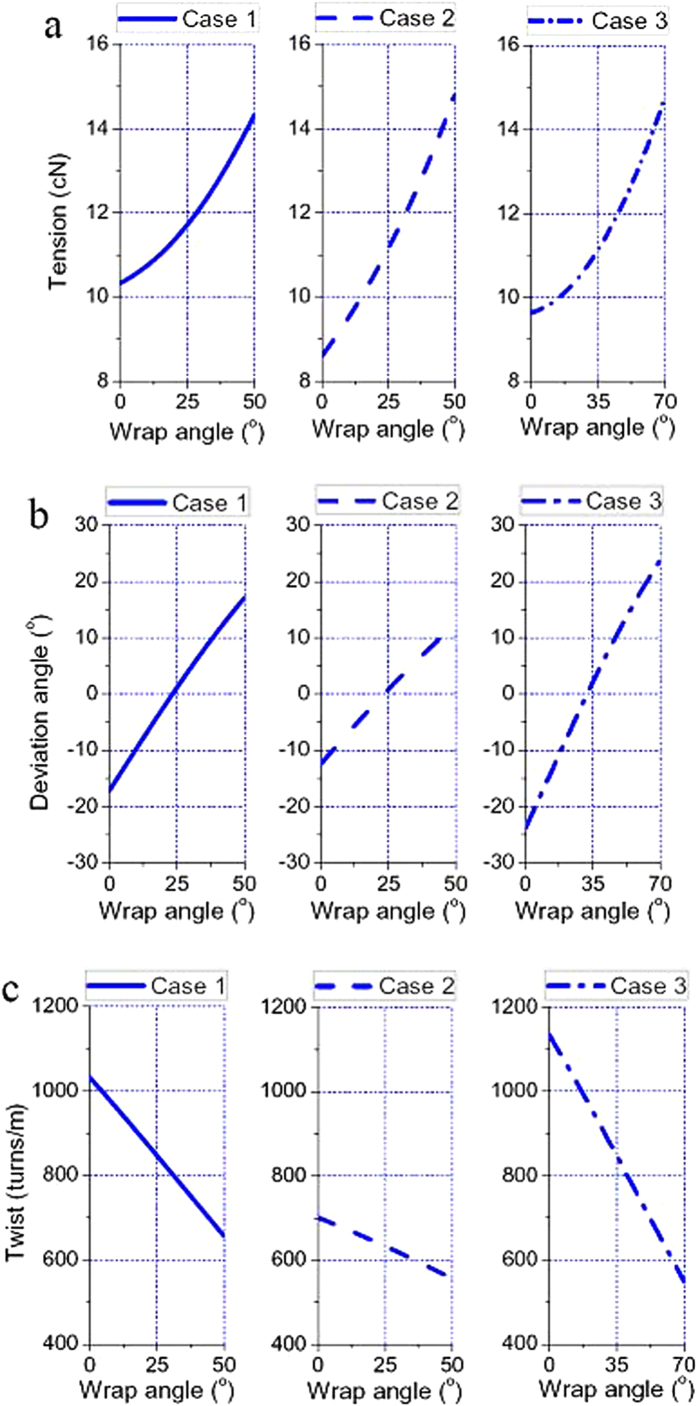
Yarn dynamic performances on the moving surface. (**a**) Tension distributions for three cases. (**b**) Distributions of deviation angle for three cases. (**c**) Twist distributions for three cases.

Figure 4a displays the simulated results of distributions of yarn tension on the moving surface against the wrap angle for three cases. Generally, the yarn tension increases linearly with the increase of wrap angle, and the tension values are raised by 38.72%, 71.58%, and 54.28% for three cases, respectively. Case 1 and 2 show that the ratio of tension in our system does not follow Euler’s equation, which is only related to frictional coefficient and wrap angle by *P*_2_/*P*_1_ = *e*^*μθ*^. In other word, tension changes in this kind of twisting system should use the treatment expressed in the first formula of equation (33).

Figure 4b plots the distributions of deviation angle against the wrap angle for the three cases. Since the length of line AB is the same as that of line CD, the deviation angle of line AB has the identical absolute value of that of line CD. Moreover, at the middle of wrap angle, 25° for case 1 and 2, and 35° for case 3, the deviation angles in all the cases are larger than zero, which means the yarn curves on the moving surface are not strictly antisymmetric.

Figure 4c depicts the distributions of twist against the wrap angle for the three cases. For all cases, the twist decreases greatly as the wrap angle increases, therefore the yarn undergoes an untwisting process on the moving surface. In case 1 and 2, the twists are reduced by 36.43% and 20.79%, respectively, while in case 3, a higher level of reductions of 51.93% is recorded. This reduction in case 3 may be caused by the increased wrap angle, which increases the contact friction between the yarn and the moving surface.

### Twist efficiency, coefficients of twist trapping and congestion

From equations (27, 28, 29), twist efficiency, coefficients of twist congestion and trapping can be calculated. The relationships between the twisting coefficients and four influencing factors, that is, yarn twist, tension, speed ratio, and wrap angle, were revealed.

#### The effect of yarn twist

Five twist levels of 458, 563, 656, 794 and 901 turns/m at the twister D were used. The other parameters are listed in Table 2. It was noted that the delivery speed of the yarn varied with the different twist levels because of the machine setting. Moreover, due to the effect of twist contraction, the tension |**P**|_*AB*_ slightly increases with the increment of twist level.

**Figure 5:**
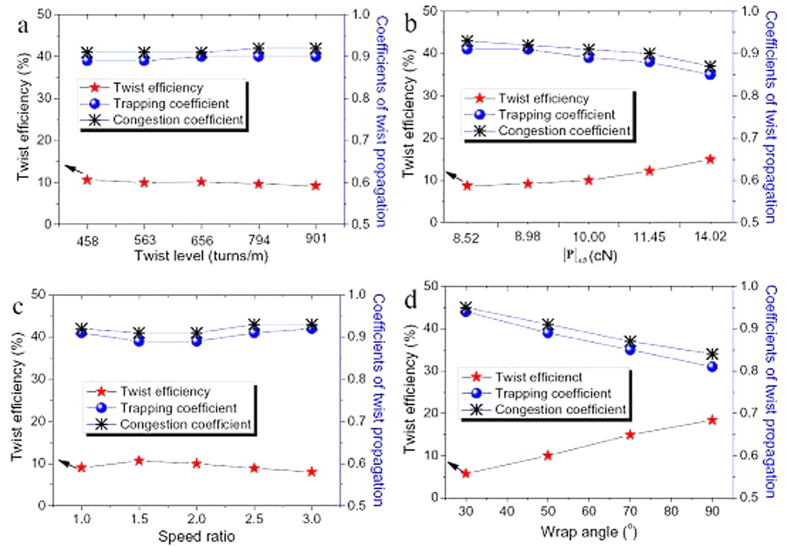
Twist efficiency and coefficients of twist trapping and congestion by univariate experiment. (**a**) Different twist levels. (**b**) Different tension levels. (**c**) Different speed ratio. (**d**) Different wrap angle.

Figure 5a plots the twist efficiency, coefficients of twist trapping and congestion against twist levels. The twist efficiency of the moving surface shows a mild descending trend from 10.66% to 9.25% when the yarn twist doubles, while the trapping and congestion coefficients maintain around 0.9 for various twist level. It is evident that the twist efficiency and the propagation coefficients are not affected by the change of yarn twist. In addition, the low twist efficiency and high propagation coefficients are caused by the relative motion of the yarn and the moving cylinder. In most circumstances, the yarn slips on the moving cylinder, therefore the level of twist blockage is low and the propagation is high.

#### The effect of yarn tension

Five different tension levels ranging from 14.02 to 8.52 cN at zone AB were used with other parameters listed in Table 2. The number of twist in zone CD was set at 560 turns/m as compared with a 3% difference of the measured twist.

Figure 5b displays the twist efficiency, propagation coefficients of twist trapping and congestion with regard to various tension levels. It implies that the yarn tension has a large impact on the twist efficiency. The twist efficiency was increased greatly from 8.70% to 14.95% when the tension level rises from 8.52 to 14.02 cN. Besides, the propagation coefficients of twist trapping and congestion decrease with the increase of the tension. A high yarn tension results in a high normal force acting on the moving cylinder, thus achieves a high twist efficiency and low propagation coefficients.

#### The effect of speed ratio

Five different speed ratios from 1 to 3 were studied with other parameters for the experiment listed in Table 2. A high value of speed ratio results in a high tension, because the moving cylinder drags the yarn in *v*_*b*_ direction heavier, leading to a high tension level.

As shown in Fig. 5c, when the speed ratio increases from 1 to 3, the number of twist in zone AB rises continuously by 45%. Nevertheless, the twist efficiency does not show the same trends. With the increasing of speed ratio, the twist efficiency of the moving surface goes up at first, and then reaches the maximum values at the speed ratio of 1.5. then decreases with further increasing of the speed ratio. On the contrary, the propagation coefficients of twist trapping and congestion exhibit a reverse trend, reaching the bottom values at the speed ratio of 1.5.

#### The effect of wrap angle

Figure 5d illustrates near linear relationships can be obtained between the three coefficients and the wrap angle. The twist efficiency is increased by 3.21 times as the wrap angle triples. By contrast, the propagation coefficients decrease from 0.91 and 0.92 to 0.81 and 0.84, respectively. It can be concluded that the twist trapping and congestion have the same trends with the change of system parameters, which means the moving cylinder blocks the both-side twist propagation with a similar amount. Moreover, the twist efficiency displays a reverse trend compared to that of propagation of twist trapping and congestion.

### Torsional rigidity

**Figure 6:**
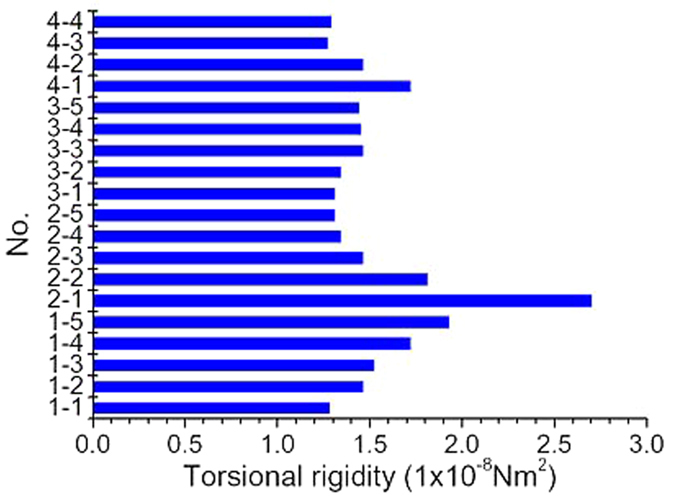
Calculated torsional rigidities for different cases.

Yarn torsional rigidity is the ratio of the applied torque and angle of twist, which is influenced by yarn geometry, twist, tension, and processing history, etc. Figure 6 displays the calculated torsional rigidities for different cases, showing significant variations. In case 2–1, the torsional rigidity reaches as high as 2.70 × 10^−8^ Nm^2^ due to the high tension value, and in other cases, the value ranges from 1.3 to 2.0 × 10^−8^ Nm^2^. Therefore, the torsional rigidity can not be set as a constant known value for simulation because it changes with the system parameters.

## Conclusions

This paper has developed a validated theoretical model of flexible yarn dynamics on a moving rigid cylinder. It was found that wrap angle and yarn tension have large influence on the twisting process, and the yarn torsional rigidity varies with the system parameters. The current work provides a theoretical foundation for applications in the fields of conventional textiles and smart materials. The treatment is in a general form, hence, the moving rigid cylinder can be replaced by other types of twisters for practical applications.

## Additional Information

**How to cite this article**: Yin, R. *et al*. Mathematical Modeling of Yarn Dynamics in a Generalized Twisting System. *Sci. Rep.* **6**, 24432; doi: 10.1038/srep24432 (2016).

## Supplementary Material

Supplementary Information

## Figures and Tables

**Table 1:** Parameters for case study and results of simulated values compared with experimental observations.

Case	v (m/s)	φ (^o^)	T_CD_ (turns/m) [CV%]	|P|_AB_ (cN) [CV%]	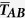 [CV%]	R_0_ (mm)	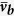	μ	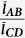
1	0.24	50	656 [15.97]	10.33 [8.58]	1.57 [14.47]	0.08	2	0.81	1
2	0.27	50	556 [13.49]	8.62 [7.94]	1.26 [12.96]	0.08	1	0.81	1
3	0.27	70	549 [15.10]	9.58 [9.32]	2.08 [13.13]	0.08	2	0.81	1
Case		|P|_*CD*_			*θ*_*AB*_			*θ*_CD_	
	M [CV%]	S	E (%)	M [CV%]	S	E (%)	M [CV%]	S	E (%)
1	14.30 [8.32]	14.33	0.21	−15.65 [11.67]	−17.15	9.58	16.23 [12.43]	17.15	5.67
2	13.62 [9.81]	14.79	8.59	−11.32 [8.76]	−12.42	9.72	11.86 [15.87]	12.42	4.51
3	15.08 [7.43]	14.78	1.99	−22.73 [11.68]	−23.77	4.58	−22.42 [11.32]	23.77	6.02

Note that M, S and E represent the measured value, simulated value, and error, respectively.

**Table 2:** Parameters of different univariate experiment.

No.	v (m/s)	φ (^o^)	*T*_*CD*_ (turns/m) [CV%]	|P|_AB_ (cN) [CV%]	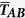	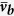
1–1	0.33	50	458 [17.92]	9.62 [8.67]	1.91 [15.70]	2.0
1–2	0.27		563 [18.23]	10.00 [8.63]	1.67 [14.57]	
1–3	0.24		656 [15.97]	10.33 [8.58]	1.57 [14.47]	
1–4	0.21		794 [14.72]	10.74 [8.66]	1.43 [14.91]	
1–5	0.18		901 [14.55]	11.20 [9.40]	1.35 [14.22]	
2–1	0.27	50	561 [14.56]	14.02 [9.09]	2.06 [15.41]	2.0
2–2			568 [16.15]	11.45 [8.54]	1.83 [15.32]	
2–3			563 [18.23]	10.00 [8.63]	1.67 [14.57]	
2–4			578 [16.40]	8.98 [8.78]	1.59 [16.54]	
2–5			543 [15.89]	8.52 [9.60]	1.60 [13.67]	
3–1	0.27	50	556 [13.49]	8.62 [7.94]	1.26 [12.96]	1.0
3–2			566 [15.17]	9.84 [8.42]	1.51 [14.70]	1.5
3–3			563 [18.23]	10.00 [8.63]	1.67 [14.57]	2.0
3–4			570 [16.02]	10.32 [9.27]	1.75 [12.02]	2.5
3–5			552 [17.26]	11.12 [9.65]	1.85 [14.07]	3.0
4–1	0.27	30	534 [15.14]	11.20 [8.89]	1.39 [15.43]	2.0
4–2		50	563 [18.23]	10.05 [8.76]	1.67 [14.57]	
4–3		70	549 [15.10]	9.58 [9.32]	2.08 [13.13]	
4–4		90	562 [15.44]	9.73 [8.87]	2.35 [15.11]	
