# Supplementary material for: Mathematical Modeling of Yarn Dynamics in a Generalized Twisting System
Source: Sci Rep. 2016 Apr 15;6:24432. doi: 10.1038/srep24432 (PMC4832211; doi:10.1038/srep24432)
Supplement: Supplementary Information [file srep24432-s1.doc]

Mathematical Modeling of Yarn Dynamics in a Generalized Twisting System

R Yin, X M Tao* and B G XU

**Measurement of yarn tension**

Before online measurement of yarn tension, the strain sensor must be calibrated by corresponding material to be measured. According to the instruction provided by the manufacture, 50cm of measured material was threaded into the sensor mounted with a stand and sensor clamping device with a suitable calibration weight, and then moved the measured material slowly and smoothly, at a speed approximates 0.5 m/s. The process was repeated thrice and the results were recorded. In the calibration process, yarn tensions with various dead weights were tested by the tension meter and the results were shown in Supplementary Figure S1. The measuring value covers all the operational range in the experiment. As displayed in Supplementary Figure S1, the measured data are very close to the real weight and the results can be expressed by the linear regression formula as

*Y=1.001X-0.081* (S1)

The correlation coefficient is 0.999.

For online tension measurement, the sampling frequency was 1000Hz and 3000 data was gathered for each measurement, as shown in Supplementary Figure S2.

**Supplementary Figure S1** Calibration of yarn tension

**Supplementary Figure S2** Measured yarn tension

**Measurement of yarn twist and deviation angle**

As shown in Supplementary Figure S3, high-speed photography technique was applied to capture images of yarn twist, and the system is composed of a high-speed camera, a light source and tripod frames. The tripod frame of the high-speed camera was used to locate the direction of camera lens perpendicular to the yarn profile, which ensures the precision of the measurement. The light was used for providing sufficient illumination during the shooting process.


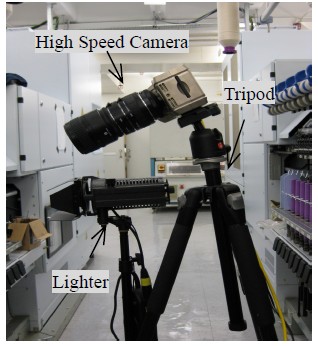


**Supplementary Figure S3** A high-speed photography system

In order to obtain the twist from images, a black-white yarn was adopted, and a scale paper of 1 mm x 1 mm size was put beneath the yarn to calculate the real length of one twist turn, as shown in Supplementary Figure S4. The yarn twist can be derived by *T=1/h* , where *T* represents the number of twist turns per unit length, *h* is the length of one turn of twist. Sixty images were used for the computation of yarn twist at each boundary. And for each image, three readings were extracted, generating 150 raw data.


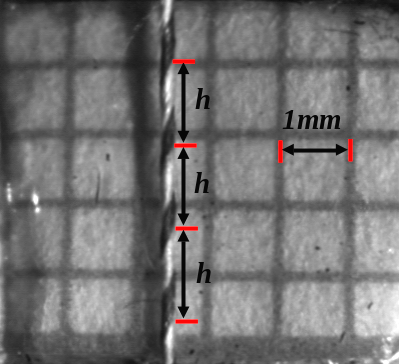


**Supplementary Figure S4** Determination of yarn twist from image

Before measurement of the twist, the high-speed camera system was calibrated. Six twist levels were used for calibration and images of the twist were captured under a resolution of 512 × 512 pixels with a sampling frequency of 1000 frames per second. The measured values were compared with the results by using standard testing method ASTM D1423-02. As shown in Supplementary Figure S5, twist measured by the high-speed photography method is approximately linear against the benchmark results by the standard method, which implies that the high-speed photography method can provide adequate accuracy and reliability for twist measurement. The relationship can be expressed by the linear regression equation as

*Y=0.915X+9.018* (S2)

The correlation coefficient is 0.999.

**Supplementary Figure S5** Calibration of yarn twist

By using the same principle, the deviation angle at two boundaries can be measured by the high-speed photography method. One hundred images were used for the calculation of deviation angle at each boundary.
